# A Survey of Venous Thromboembolism (VTE) Prophylaxis in Obstetrics Patients in Iran 

**Published:** 2019-03

**Authors:** Majid Mokhtari, Khadijeh Nasri, Fatemeh Tara, Elahe Zarean, Sedigheh Hantoushzadeh, Mehrnaz Radmehr, Maryam Kashanian

**Affiliations:** 1 Department of Internal Medicine, Shahid Beheshti University of Medical Sciences, Imam Hosein Hospital, Tehran, Iran; 2 Department of Obstetrics and Gynecology, Arak University of Medical Sciences, Arak, Iran; 3 Department of Obstetrics and Gynecology, Mashhad University of Medical Sciences, Ommol Banin Teaching Hospital, Mashhad, Iran; 4 Department of Obstetrics and Gynecology, Isfahan University of Medical Sciences, Alzahra Teaching Hospital, Isfahan, Iran; 5 Maternal-Fetal Medicine Research Center, Tehran University of Medical Sciences, Imam Khomeini Teaching Hospital, Tehran, Iran; 6 Department of Obstetrics and Gynecology, Milad Hospital, Tehran, Iran; 7 Department of Obstetrics and Gynecology, Iran University of Medical Sciences, Akbarabadi Teaching Hospital, Tehran, Iran

**Keywords:** Venous Thrombo-embolism, Obstetric Population, Deep Venous Thrombosis, VTE Prophylaxis, Pulmonary Embolism

## Abstract

**Objective:** The purpose of the present study was a survey of venous thromboembolism (VTE) prophylaxis in obstetrics patients in Iran.

**Materials and methods:** A national, multicenter, non-interventional, prospective study was performed on 1000 women at 11 different parts of Iran. Primary outcome was to assess the situation of VTE prophylaxis in pregnant and postpartum women and the secondary outcome was risk stratification in obstetrics patients and to evaluate the guideline adherence in physician’s practice of VTE prophylaxis.

**Results:** 1,036 women entered the final analysis. The three main VTE risk factors before hospitalization were BMI > 30 kg/m2, history of oral contraceptive (OCP) use, and the age over 35.VTE risk factors upon enrollment were detected in 780 (75.28%) patients. 219 women (28.07%) were deemed eligible for drug prophylaxis, however, only 37 women (17%) received it. A total of 113 (10.9%) patients received VTE prophylaxis, of which 76 (67.25%) women had no clear indications. Concordance between theory and practice was detected with a Cohen’s Kappa coefﬁcient to be 0.74 (p < 0.001), which fell within “good agreement”. Multivariate analysis for association between VTE prophylaxis and VTE risk factors showed that history of VTE [OR = 9.06 (CI 95% 1.16 – 70.8) p = 0.036] was the most frequent risk factor for receiving VTE prophylaxis followed by obesity (BMI > 30 Kg/m2); [OR = 3.74 (CI 95% 1.79 – 5.69), p = <0.001], multiple pregnancy [OR= 2.81 (CI 95% 1.70 – 4.64), p = < 0.001] and age > 35 years; [OR =1.09 (CI 95% 0.82 – 1.21), p = 0.026]. Varicose Veins [OR= 0.22 (CI 95% 0.56 – 0.87), p = 0.031], PROM / PPROM [OR= 0.33 (CI 95% 0.12 – 0.91), p = 0.032] and history of using OCP [OR= 0.36 (CI 95% 0.24 – 0.53), p = < 0.001] were the most missed risk factors for receiving VTE prophylaxis respectively.

**Conclusion:** History of VTE, obesity, multiple pregnancy and age > 35 years were the most frequent risk factors for receiving VTE prophylaxis and varicose veins, PROM / PPROM and history of using OCP were the most missed risk factors for receiving VTE prophylaxis.

## Introduction

Deep venous thrombosis (DVT) and pulmonary embolism (PE), collectively known as venous thromboembolism (VTE), are leading causes of morbidity and mortality, especially in hospitalized patients. This problem may occur in pregnant women because pregnancy is associated with an increased clotting potential, decreased anticoagulant activity and decreased fibrinolysis that generally make a pregnant woman at risk for VTE (1). Also, the thrombotic potential of pregnancy is exacerbated by venous stasis in the lower extremities due to the compression of the inferior vena cava and pelvic veins by the enlarging uterus, hormone mediated increase in venous capacitance which enhances the risk of DVT and subsequent VTE (1). Pulmonary embolism is the most important direct cause of maternal death in the UK and the United States and is the second most common cause of maternal death overall (11% of maternal deaths) (2, 3). VTE complicates approximately 1 in 1600 births (3), and 79% of the women who died from pulmonary embolism between 2003 and 2005 had identifiable risk factors (4).

Many antenatal VTE events occur in the first trimester and therefore prophylaxis, if given, should begin early in pregnancy (5), however, the highest risk period for VTE, and pulmonary embolism in particular, is during the postpartum period and caesarean section is a significant risk factor but women having vaginal deliveries are also at risk (6, 7). A cohort study from Rochester, Minnesota, showed that the annual incidence of VTE was five times higher in the postpartum period (5).

Pulmonary embolism can be prevented with appropriate thrombo-prophylaxis after careful VTE risk assessment. Anticoagulant therapy is frequently used during pregnancy for the prevention and treatment of VTE (8, 9).

The aim of this study was to assess the VTE prophylaxis in pregnant and postpartum women as a multicenter study at five large provinces of Iran, because, there were no published information on the extent of VTE prophylaxis.

## Materials and methods

A national, multicenter, non-interventional, prospective study was performed between Aug 2013 to Dec 2013, for assessing VTE prophylaxis and VTE risk factors in pregnant women. This pilot study was conducted for the first time in Iran. So, it was considered to evaluate 1000 patients regarding antenatal and postnatal care for VTE prophylaxis from 11 hospitals across the country with about 100 patients at each site. The study was finally conducted at 11 centers supervised by 11 investigators. Primary outcome was to assess the situation of VTE prophylaxis in pregnant and postpartum women in these selected study centers and the secondary outcome was to appraise VTE risk assessment and risk stratification in patients and to evaluate the guidelines adherence in physician’s practice of VTE prophylaxis. The study was approved by the ethic committee of Tehran University of Medical Sciences. Hospitals were selected randomly in five provinces. The investigators included patients sequentially at each site for their obstetric evaluation. After obtaining informed consents, pregnant and postpartum women who agreed to participate in the survey and needed hospitalization, were included in the study. Patients were excluded if they had been admitted for VTE treatment; were on anticoagulation therapy prior to admission in the hospital, and in the case of missing hospital chart. 

A specially designed clinical record forms (CRF) was used for data collection, completed by participating principal investigators or their delegated staff. Data regarding demographic characteristics, current clinical status, VTE risk factors, bleeding risk factors, and VTE prophylaxis were collected. The data quality control was performed in sites where the study was performed for at least 10% of the enrolled patients.Demographic data such as age, body mass index (BMI), gravidity, parity, abortions, gestational age, complications during pregnancy and postpartum issues were collected. Data regarding VTE risk factors such as prior history of VTE, thrombophilia, family history of VTE, smoking, assisted reproductive therapies (ART), preeclampsia/eclampsia, and multiple pregnancies were similarly obtained.

Baseline characteristics, including demographics, medical history, nature, duration, and severity of the disease, comorbidities, and current treatment were summarized into counts of non-missing data.Mean, standard deviation, minimum, maximum, and median with a 95% confidence interval (CI) were calculated for categorical data.

## Results

A total of 1,050 patients were enrolled in the survey. 14 patients were excluded, 9 for missing informed consent, and 5 for not meeting eligibility criteria. 

**Table 1:** The characteristics of 1036 women

**Variables**			**Missing value**
Age of patient (years) (Mean± sd)		28.18 ± 5.7	6
BMI before gestation (kg/m2) (Mean± sd)		24.3 ± 4.9	9
BMI on enrolment (kg/m2) (Mean± sd)		29.1 ± 5.1	9
Age Groups [n (%)]	< 20	72 (6.9%)	6
	20 – 30	609 (58.8%)	
	30 – 40	326 (31.5%)	
	> 40	23 (2.2%)	
BMI Groupson enrolment kg/m2 [n (%)]	Underweight (< 18.5)	6 (0.6%)	9
	Normal (18.5 – 25)	219 (21.2%)	
	Overweight (25.1 – 30)	385 (37.1%)	
	Obese (30.1 – 40)	395 (37.2%)	
	Morbid (> 40)	22 (2.1%)	
Gravidity (Mean± sd)		2 ± 1.1	1
Parity (Mean± sd)		1 ± 0.9	0
Number of Abortion (Mean± sd)		0 ± 0.6	1
Gestational age at recruitment (Weeks) (Mean± sd)		34.8 ± 7.4	4
Labor in past 12 months [n(%)]		5 (0.5%)	0
Abortion in past 12 weeks [n(%)]		6 (0.6%)	0

Data on 1,036 patients entered the final analysis. The baseline characteristics of the women were shown in table 1. The most frequent reasons for hospitalization were emergency and elective cesarean section and vaginal delivery in 335 (32.3%) and 290 (28%) participants, respectively. The three main VTE risk factors before hospitalization were BMI > 30 kg/m2 in 417 (40.2%), history of oral contraceptive (OCP) use in 146 (14%), and the age over 35 in 129 (12.5%) (Table 2).

**Table 2:** VTE risk factors before admission in hospital (n = 1036)

**Risk factors for VTE at enrolment**	**n**	**%**
BMI (>30 Kg/m^2^)	417	40.25
History of OCP	146	14.1
Age (>35 years)	129	12.5
Long Distance travel	129	12.5
Multiple pregnancy	113	10.9
History of Hospitalization	78	7.5
Pre-eclampsia	77	7.4
PROM / PPROM		
< 24 hour	74	7.1
> 24 hour	21	2.0
Immobility	59	5.7
Parity (> 3)	61	5
Post-Partum Hemorrhage	51	4.9
Assisted reproductive therapy	41	4.0
Dehydration /OHSS	30	2.9
History of VTE	19	1.8
Current Systemic infection	6	0.6
Previous VTE	5	0.5
Smoking	4	0.4
Varicose Veins	13	0.3

VTE risk factors upon enrollment were detected in 780 (75.3%) patients. 355 women (45.5%) had one, and 11 women (1.4%) had 6 or more identifiable risk factors (Table 3). Patients in the low, moderate, and high VTE risk categories were 559 (71.6%), 220 (21.2%), and 1 (0.1%), respectively.

**Table 3:** Frequency of VTE Risk Factors by numbers (n= 1036)

**Risk factors by number**	**n**	**%**
No Risk factor	256	24.7
1 Risk factor	355	34.3
2 Risk factors	228	22.0
3 Risk factors	125	12.1
4 Risk factors	47	4.5
5 Risk factors	14	1.4
6 Risk factors	8	0.8
7 Risk factors	3	0.3

**Figure 1:**
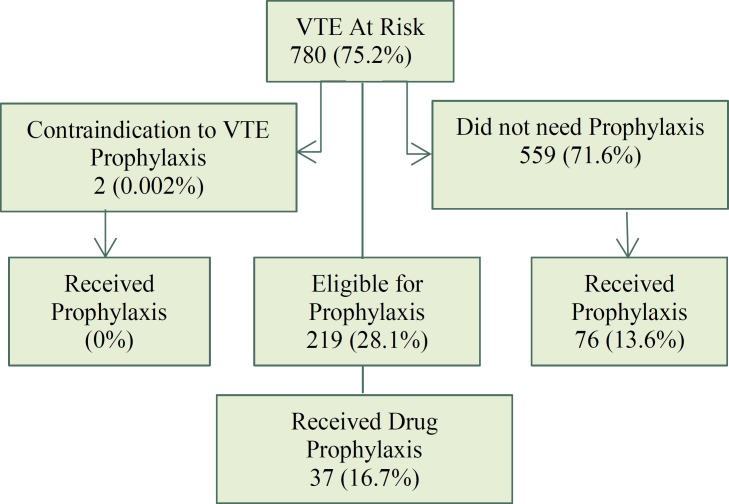
Patients at risk for VTE and receiving prophylaxis

Of 780 women (75.2%) at risk for VTE, 221 women (28.28%) were deemed eligible for drug prophylaxis. Of the 221 patients, 2 (1.7%) had a contraindication for receiving it, and only 37 women from 219 women (17%) who needed to administer VTE prophylaxis received it (Figure 1). A total of 113 (10.9%) patients received VTE prophylaxis, of which 76 (67.25%) women had no clear indications. The analysis of concordance between VTE prophylaxis theory and practice showed that from 815 (78.66%) participants who did not require any VTE prophylaxis, 76 women (10%) received it, and from 221 women (28.1%) who were eligible for it, only 37 women (17%) received it. 

**Table 4:** Concordance between Theory and Practice (n = 1036-2* =1034)

	**Should not receive VTE prophylaxis** **n = 815**	**Should receive VTE prophylaxis ** **n = 219**	**Total**
Did not receive VTE prophylaxis	739 (71.33%)	182 (17.76%)	921 (89.09%)
Received VTE prophylaxis	76 (7.33%)	37 (3.57%)	113 (10.90%)
Total	815 (78.66%)	219 (21.33%)	1034

* contraindication to VTE Prophylaxis

Coherence between theory and practice has been detected with a Cohen’s Kappa coefﬁcient of 0.74 (p < 0.001), which fell within “good agreement” (Table 4).

Multivariate analysis for association between VTE prophylaxis and VTE risk factors showed that history of VTE [OR = 9.06 (CI 95% 1.16 – 70.8) p = 0.036] was the most frequent risk factor for receiving VTE prophylaxis followed by obesity (BMI > 30 Kg/m2); [OR = 3.74 (CI 95% 1.79 – 5.69), p = <0.001], multiple pregnancy [OR= 2.81 (CI 95% 1.70 – 4.64), p = < 0.001] and age > 35 years; [OR =1.09 (CI 95% 0.82 – 1.21), P= 0.026]. Varicose Veins [OR= 0.22 (CI 95% 0.56 – 0.87), P= 0.031], PROM / PPROM [OR= 0.33 (CI 95% 0.12 – 0.91), P= 0.032] and history of using OCP [OR= 0.36 (CI 95% 0.24 – 0.53), p = < 0.001] were the most missed risk factors for receiving VTE prophylaxis respectively (Table 5).

## Discussion

1,036 women were evaluated in this multicenter study in Iran. The three main VTE risk factors before hospitalization were BMI > 30 kg/m2, history of oral contraceptive (OCP) use and age over 35.However, only 17% of women who wereeligible for drug prophylaxis received it. Concordance between theory and practice has been detected to show “good agreement”. History of VTE was the most frequent risk factor for receiving VTE prophylaxis followed by obesity, multiple pregnancy and age > 35 years. Varicose veins, PROM / PPROM and history of using OCP were the most missed risk factors for receiving VTE prophylaxis. This study highlighted a serious concern for a national guideline for VTE prophylaxis which finally was provided by the office of maternal health at the Iranianministry of health.

**Table 5:** Multivariate Analysis; Association of VTE prophylaxis and VTE risk factors

	**OR**	**95% C.I**	**P- value**
History of VTE	9.06	1.16 – 70.8	0.036*
Obesity (BMI > 30 Kg/m2)	3.74	1.79 – 5.69	<0.001*
Multiple Pregnancy	2.81	1.70 – 4.64	< 0.001*
Age > 35 years	1.09	0.82 – 1.21	0.026*
VaricoseVeins	0.22	0.56 – 0.87	0.031*
PROM / PPROM	0.33	0.12 – 0.91	0.032*
History OCP	0.36	0.24 – 0.53	< 0.001*

PROM = Premature rupture of membrane, OCP = Oral contraceptive pills

* significant

A BMI > 30 kg/m2, in prior studies was reported to be an underestimated risk factor for VTE during pregnancy. VTE in the presence of obesity carries a significant mortality risk as shown in a study from the UK between 2003 and 2005 (4).

Also, guidelines, mainly form developed countries, highlighted obesity (BMI > 30 kg m^2^) and prior VTE, either with or without other VTE risk factors, as important risk factors in the development of VTE during pregnancy and childbirth (2, 10). Therefore, losing weight before pregnancy and reaching the ideal weight should be encouraged at pre-conception counselling (11). Also, weight management during antenatal period, in order to prevent excessive weight gain should be considered. In the present study, the most common risk factor for VTE was obesity with a BMI > 30 kg/m^2^.History of taking oral contraceptive pills was the second most frequent risk factor for VTE in this study, however, it was the most frequent ignored risk factor in VTE risk assessment too. Therefore, a check list of important risk factors for VTE should be available in each obstetric unit (10). Age of more than 35 years and long haul travelling were the other risk factors for VTE, which could be disseminated to patients in order for them to make informed choices when planning for their pregnancy (12).

A wide discrepancy was observed when comparisons were made between patients who had VTE risk stratification and prophylaxis management. The number of women who were deemed to have no VTE risk factors were overestimated, and consequently those with low and moderate VTE risk factors were underestimated. These discrepancies were culminated to the inappropriate application of VTE prophylaxis for women. Therefore, there is a need for guidelines and checklists for better evaluation of patients who might be at risk (10).

## Conclusion

As a conclusion, VTE risk prophylaxis in the obstetric population needed improvement in Iran. History of VTE, obesity, multiple pregnancy and age > 35 years were the most frequent risk factors for receiving VTE prophylaxis and varicose veins, PROM / PPROM and history of using OCP were the most missed risk factors for receiving VTE prophylaxis.
